# Regorafenib as a potential drug for severe COVID‐19: inhibition of inflammasome activation in mice

**DOI:** 10.1002/2211-5463.70002

**Published:** 2025-02-03

**Authors:** Ju Hwan Jeong, Sun‐Ok Kim, Seong Cheol Min, Eung‐Gook Kim, Min‐Suk Song, Eun‐Young Shin

**Affiliations:** ^1^ Department of Microbiology, Chungbuk National University College of Medicine and Medical Research Center Chungbuk National University Hospital Cheongju Republic of Korea; ^2^ Department of Biochemistry, Chungbuk National University College of Medicine and Medical Research Center Chungbuk National University Hospital Cheongju Republic of Korea

**Keywords:** infection, inflammasome, K18‐hACE2, regorafenib, SARS‐CoV‐2

## Abstract

SARS‐CoV‐2 infection can lead to severe COVID‐19, particularly in elderly individuals and those with compromised immunity. Cellular senescence has been implicated as a key pathogenic mechanism. This study investigated the therapeutic potential of regorafenib, a previously characterized senomorphic drug, for severe COVID‐19. SARS‐CoV‐2 virus‐infected K18‐hACE2 mice, overexpressing the human ACE2 receptor, exhibited 100% mortality by 10 days post infection. Regorafenib treatment significantly improved survival rates, approximately 43% remaining alive. Mechanistically, regorafenib effectively suppressed type I and II interferon and cytokine signaling. Notably, regorafenib inhibited NLR family pyrin domain containing 3 (NLRP3) inflammasome activation, a key driver of the cytokine storm associated with severe COVID‐19. Our findings elucidate the molecular mechanisms underlying therapeutic effects of regorafenib and suggest its potential use as a promising treatment option for severe COVID‐19.

## Graphical abstract

Regorafenib treatment significantly enhanced survival in SARS‐CoV‐2‐infected K18‐hACE2 mice. Mechanistically, it suppressed type I and II interferon and cytokine signaling through inhibiting NLRP3 inflammasome activation, a critical driver of the cytokine storm in severe COVID‐19. These findings elucidate the molecular mechanisms of regorafenib's therapeutic effects and highlight its potential as a treatment for severe COVID‐19.

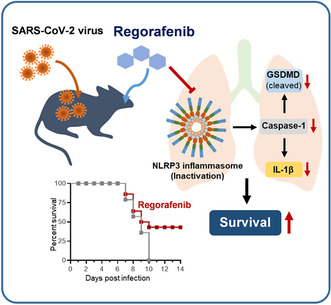

**Abbreviations**
CASP1: Caspase‐1
COVID‐19: coronavirus disease 2019
dpi: days post infection
GSDMD: Gasdermin D
hACE2: human angiotensin‐converting enzyme 2
IFN: interferon
IL: interleukin
NLRP3: NLR family pyrin domain containing 3
Reg: Regorafenib
SARS‐CoV‐2: severe acute respiratory syndrome coronavirus‐2
SASP: senescence‐associated secretory phenotype

SARS‐CoV‐2 infection induces the innate immunity in host followed by the adaptive immunity represented by the activation of T and B cells. Major components of the innate immune system against SARS‐CoV‐2 infection involve a robust interferon response, mostly type I and II interferons (IFNs), and a subsequent inflammatory response mediated by diverse cytokines and chemokines released by the innate immune cells [1]. A delayed or weak response of the innate immune system can be a cause of infection severity in COVID‐19 patients, whereas a persistently exaggerated response may induce a ‘cytokine storm’ or hyperinflammation which lead to death [2]. In this regard, to modulate the ‘cytokine storm’ or hyperinflammation, numerous translational investigations have been under way.

Recent studies have demonstrated that reducing the senescent cell burden and the inflammatory senescence‐associated secretory phenotype (SASP) by treatment with senolytic compounds improves the immune response and reduces the mortality of COVID‐19 patients [3]. Our previous study demonstrated a beneficial effect of a senomorphic drug regorafenib (Reg), an inhibitor drug of multi‐receptor tyrosine kinases, in pulmonary emphysema, a senescence‐associated chronic lung disease [4]. Reg is a Food and Drug Administration (FDA)‐approved drug for the treatment of metastatic colorectal cancer [5], and is also used for advanced gastrointestinal stromal tumors and hepatocellular carcinoma [6, 7]. Recently, there have been further reports on the antiviral potential of Reg against severe SARS‐CoV‐2 infection [8], including an anti‐cytopathic effect in Caco2 and VERO‐6 cells [9]. However, the precise mechanism by which Reg regulates SARS‐CoV‐2 infection remains unclear.

Our previous studies identified the major mode of action of Reg as its anti‐SASP effect, which reduces the levels of cytokines such as interleukin‐1β (IL‐1β), tumor necrosis factor‐α (TNF‐α), IL‐6 and IL‐8 [4, 10]. These results implicate the therapeutic potential of Reg in acute lung injury and acute respiratory distress syndrome (ARDS), including COVID‐19. To further investigate this, we therefore examined the therapeutic effect of Reg on COVID‐19 by employing K18‐hACE2 transgenic mice (hACE2 mice) that overexpress human angiotensin‐converting enzyme 2 (ACE2) receptors driven by cytokeratin‐18 (K18) gene promoters, a cellular entry point of the SARS‐CoV‐2 virus [11, 12]. This study aims to evaluate efficacy of Reg in mitigating SARS‐CoV‐2‐induced pathologies and to elucidate its underlying mechanisms of action.

## Materials and methods

### Mouse

Eight‐week‐old female B6. Cg‐Tg (K18‐ACE2) 2Prlmn/J mice (stock# 034860) were acquired from the Jackson Laboratory (Bar Harbor, ME, USA) and housed in separate groups for SARS‐CoV‐2 infection experiments. The mice were housed under a 12‐h light/dark cycle with free access to a standard laboratory diet and bedding (SAMTAKO, Osan, Republic of Korea). The physical condition of the animals, including body weight, activity levels, and coat condition was monitored daily for signs of illness or distress. Humane endpoints were established to minimize suffering, and euthanasia was performed when mice exhibited severe clinical symptoms, such as a body weight reduction of 25% or more from their initial weight. Euthanasia was conducted using CO_2_ inhalation in accordance with approved guidelines. Regarding mortality, no mice perished from causes other than SARS‐CoV‐2 infection. The study received approval from the Institutional Animal Care and Use Committee (IACUC) of Chungbuk National University, Republic of Korea (CBNUA‐1632‐21‐02). All procedures involving animals were carried out in strict accordance with the guidelines of Chungbuk National University, the Ministry of Food and Drug Safety, and the Korea Disease Control and Prevention Agency. The SARS‐CoV‐2 infection experiments were conducted in a Biosafety Level 3 (BSL3+) laboratory facility at Chungbuk National University.

### Protective efficacy evaluation of SARS‐CoV‐2 infected mice

On day 0, mice were anesthetized with isoflurane inhalation and intranasally inoculated with a 50 μL mixture containing 5 MLD50 of SARS‐CoV‐2 virus (β‐CoV/Korea/KCDC03/2020, S clade) to evaluate the protectivity of SARS‐CoV‐2 infection. Regorafenib (Reg, 5 mg·kg^−1^) was orally administered once daily on days 3, 4, and 5 days post infection (dpi) [4, 10]. SARS‐CoV‐2 infection experiments were conducted for each group to assess weight loss and survival (*N* = 7) as well as viral replication assessment in lung tissue (*N* = 6). Mice were carefully monitored for changes in body weight and survival until 14 dpi. If any of the mice experienced a ≥25% loss of body weight compared to pre‐infection, they were humanely euthanized.

### RT‐qPCR analysis

RNA was extracted using a RNeasy Mini Kit (QIAGEN, Hilden, Germany) from homogenized mice lung tissue. RT‐qPCR were conducted using a Bio‐Rad Real‐Time PCR system (Bio‐Rad, Hercules, CA, USA) and TOPreal Probe RT‐qPCR Kit (Enzynomics, Daejeon, Republic of Korea). The signal transducer and activator of transcription 1 (*Stat1*, Mm.PT.58.23792152), *Stat2* (Mm.PT.58.8014151), *Tnf‐α*, (Mm.PT.58.28810479), and chemokine (C‐C motif) ligand 3 (*Ccl3*, Mm.PT.58.29283216) immune‐modulating genes were quantified by pre‐designed detection primer (IDT, Corvallis, IA, USA). RT‐qPCR analysis was performed on lung tissues obtained from three mice per group.

### RNA‐sequence analysis

Total RNAs were isolated from lung tissues using Trizol reagent (Thermo Fisher Scientific, Inc., Waltham, MA, USA). RNA quality was assessed by TapeStation4000 System (Agilent Technologies, Amstelveen, The Netherlands). In addition, RNA quantification was performed using an ND‐2000 Spectrophotometer (Thermo Fisher Scientific, Inc., Waltham, MA, USA). The libraries were prepared from total RNA using the NEBNext Ultra II Directional RNA‐Seq Kit (NEW ENGLAND BioLabs, Inc., Ipswich, MA, USA). mRNA was isolated using the Poly(A) RNA Selection Kit (LEXOGEN, Inc., Vienna, Austria), and synthesized to the cDNA and amplified by PCR. Subsequently, libraries were checked to evaluate the mean fragment size using the TapeStation HS D1000 Screen Tape (Agilent Technologies, Amstelveen, The Netherlands). Library quantification was performed using a StepOne Real‐Time PCR System (Thermo Fisher Scientific, Inc.). High‐throughput sequencing was performed as paired‐end 100 sequencing using a NovaSeq 6000 (Illumina, Inc., San Diego, CA, USA). A quality control of raw sequencing data was performed using FastQC. Adapter and low quality reads (<Q20) were removed using FASTX_Trimmer and BBMap. Then, the trimmed reads were mapped to the reference genome using TopHat [13]. Gene expression levels were estimated using FPKM (Fragments Per kb per Million reads) values by Cufflinks [14]. The FPKM values were normalized based on the TMM + CPM method using EdgeR within R. Data mining and graphic visualization were performed using ExDEGA (Ebiogen Inc., Seoul, Korea) and Toolbox software. RNA‐seq analysis was performed on lung tissues obtained from five mice per group.

### Virus, cell culture and cell differentiation

Vero E6 cells (African green monkey kidney cells, ATCC^®^ cat# CRL‐1586™) were maintained in Dulbecco's Modified Eagle's Medium (DMEM) containing 10% fetal bovine serum (FBS), 1% antibiotic‐antimycotic solution, 110 mg·L^−1^ sodium pyruvate, 4.0 mm L‐glutamine, and 4.5 g·L^−1^ d‐glucose at 37 °C in a 5% CO_2_ incubator. The SARS‐CoV‐2 strain (β‐CoV/Korea/KCDC03/2020, S clade) was provided from the National Culture Collection for Pathogens, Republic of Korea. The virus was propagated and maintained in Vero E6 cells using DMEM supplemented with 2% FBS and 1% antibiotic‐antimycotic solution under optimal conditions of 37 °C in a 5% CO_2_ incubator. Human monocytic THP‐1 cells (ATCC, TIB‐202) were maintained in RPMI 1640 medium (LM011‐01, Welgene, South Korea) containing 10% of fetal bovine serum, 100 U·mL^−1^ penicillin, and 100 μg·mL^−1^ streptomycin, and maintained at 37 °C in a 5% CO_2_ incubator. THP‐1 cells were incubated to differentiate into macrophages with 100 nm phorbol 12‐myristate 13‐acetate (PMA; Sigma‐Aldrich,St. Louis, MO, USA) for 48 h.

### Immunofluorescence staining

Mouse lung tissues (*N* = 3 mice per group) were fixed with 10% neutral buffered formalin (NBF) solution, embedded in paraffin, and sectioned at a thickness of 4 μm. The slides were deparaffinized and processed for antigen retrieval boiling in Tris–HCl buffer (pH 10). The slides were washed with phosphate buffered saline‐T (PBS‐T, PBS containing 0.1% Tween 20) and permeabilized with 0.2% Triton X‐100 for 1 h. For blocking and antibody dilution, 3% normal donkey serum and 3% bovine serum albumin (BSA) in PBS containing 0.2% Triton X‐100 was used. The slides were incubated in the primary antibody solution overnight at 4 °C and washed three times with PBS‐T. The slides were incubated in Alexa‐Fluor labeled secondary antibody at 4 °C for 1 h, and then washed three times with PBS‐T. Positive staining signals were observed under confocal microscope (Zeiss LSM900, Germany) and analyzed using ImageJ and GraphPad Prism (version 8.4.3, San Diego, CA, USA) software.

### Western blotting analysis

Cells were lysed with 2X Laemmli sample buffer. The total proteins were then separated by SDS/PAGE and transferred onto PVDF membranes. The membranes were blocked and incubated with primary antibody overnight at 4 °C, followed by incubation with horseradish peroxidase (HRP)‐conjugated secondary antibody. Bands on the membrane were detected by ECL solution using ChemiDoc imaging system (Bio‐Rad Laboratories, Inc.).

### Antibody

Anti‐F4/80 antibody was obtained from Thermo Fisher Scientific, Inc. (Waltham, MA, USA). Anti‐SARS‐CoV‐2 nucleoprotein (NP) antibody was purchased from Sino Biological Sino Biological Inc. (Beijing, China). Anti‐NLRP3 antibody was purchased from Cell Signaling Technology (Danvers, MA, USA) for western blotting and Bioss Inc (Woburn, MA, USA) for immunofluorescence (IF) staining. Anti‐GSDMD antibody was obtained from Abcam (Cambridge, UK). IL‐1β antibody was purchased from Cell Signaling Technology (Danvers, MA, USA).

### Statistical analysis

In all data, error bars represent the mean ± standard error of the mean (SEM) from at least three independent experiments. Statistical significance was assessed using a two‐tailed Student's *t*‐test (GraphPad Prism, version 8.4.3, San Diego, CA, USA). The *P* values are indicated as follows: ns, no significance; *P < 0.05; **P < 0.01; ***P < 0.001.

## Results and discussion

### The survival of SARS‐CoV‐2 infected mice was increased by regorafenib (Reg) treatment

**Fig. 1:**
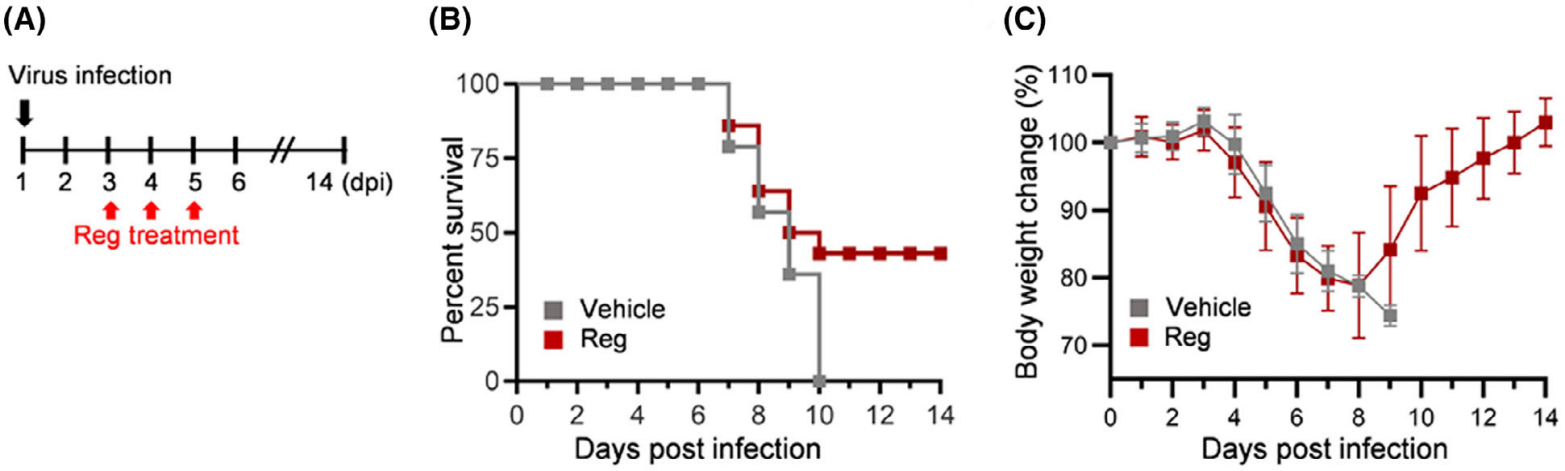
Regorafenib increases the survival rates of SARS‐CoV‐2 infected K18‐hACE2 transgenic mice. (A) Experimental scheme to define the therapeutic effect of regorafenib (Reg). hACE2 mice were infected with 5 MLD50 of SARS‐CoV‐2 (β‐CoV/Korea/KCDC03/2020, S clade) and treated with Reg (5 mg·kg^−1^) three times once daily 3 dpi to 5 dpi. (B) Survival curve. Kaplan–Meier plot of the survival of three groups (*N* = 7 for each group). Two independent experiments exhibited a similar survival curve. (C) Body weights of mice were measured daily for 14 days (*N* = 7 for each group). Mice exhibited loss ≥25% of their initial body weight were euthanized. Error bars represent standard error of the mean (SEM) for each group of mice.

hACE2 mice were infected with SARS‐CoV‐2 virus, and lung tissues were sampled as in the scheme (Fig. 1A). Regorafenib (Reg) was orally administered for 3 days starting on the 3rd day post infection (dpi). To confirm the infection of SARS‐CoV‐2 virus, lung tissues were stained for SARS‐CoV‐2 NP, a viral RNA‐binding protein, and F/80, a macrophage marker. Immunoreactivity of SARS‐CoV‐2 NP and F/80 was notably increased in SARS‐CoV‐2‐infected lungs, but decreased in response to Reg, thus confirming the viral infection (Fig. S1). The group of SARS‐CoV‐2‐infected mice without any treatment (vehicle) all succumbed to infection by 10 dpi (Fig. 1B). Reg‐treated groups showed much improved survival rates, with approximately 43% of the infected mice surviving. Additionally, body weight changes in Reg‐treated groups reached their lowest point at 8 dpi, but gradually recovered to their original weight by 14 dpi (Fig. 1C). The viral titers in the Reg‐treated mice lung tissues were comparable to those in the vehicle‐treated group, indicating that Reg does not directly inhibit SARS‐CoV‐2 (Fig. S2). Together, these results suggest a therapeutic potential of Reg as a repurposed drug for severe COVID‐19.

### Reg suppressed the activation of type I and II IFN pathway in SARS‐CoV‐2‐infected mice

**Fig. 2:**
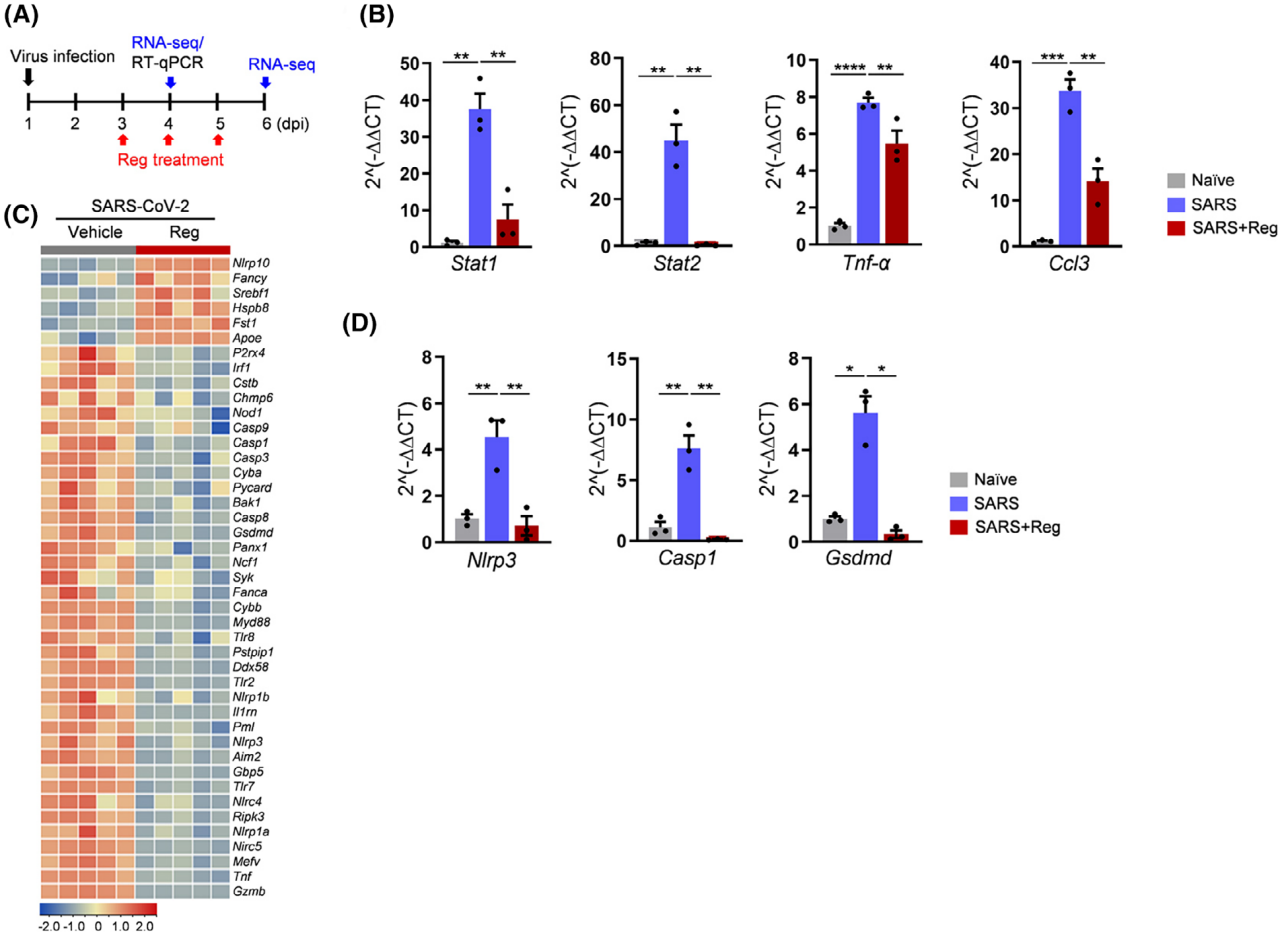
Transcriptome analysis for effect of regorafenib in SARS‐CoV‐2 infected mice. (A) Experimental scheme for RNA sequencing (RNA‐seq) analysis (B) Representative immune‐modulating genes, *Stat*1 and *Stat*2, critical transcription factors that mediate IFN signaling, *TNF‐α*, and *Ccl3* were subjected to RT‐qPCR. Naïve; SARS, SARS‐CoV‐2‐infected; SARS + Reg, Reg‐treated following SARS‐CoV‐2 infection. *N* = 3 for each group. Student's *t*‐test, *P < 0.05; **P < 0.01; ***P < 0.001. Error bars represent standard error of the mean (SEM) for each group of mice. (C) Heat map of regorafenib‐modulated inflammasome‐ and pyroptosis‐related genes at 4 dpi following SARS‐CoV‐2 infection. *N* = 5 for each group. (D) Validation of RNA‐seq analysis by RT‐qPCR. *Nlrp3* and its downstream effectors *Casp1* and *Gsdmd* were analyzed. Naïve, non‐infected; SARS, SARS‐CoV‐2‐infected; SARS + Reg, Reg‐treated following SARS‐CoV‐2 infection (*N* = 3 for each group). Student's *t*‐test, *P < 0.05; **P < 0.01; ****P < 0.0001. Error bars represent standard error of the mean (SEM) for each group of mice.

To elucidate the mechanism by which Reg exerts its beneficial effects on the survival of SARS‐CoV‐2‐infected mice, we performed a transcriptome analysis. For this purpose, as shown in Fig. 2A, hACE2 mice were treated with Reg following SARS‐CoV‐2 infection and lung tissues were sampled on 4 dpi (early) and 6 dpi (late). We carefully analyzed the alterations in type I and II IFN and cytokine‐mediated signaling related with the innate immune response because these signaling pathways are crucial for the pathogenesis of SARS‐CoV‐2 infection [11, 15]. At 4 dpi, in general, homogenous downregulation of Reg‐responsive genes was evident in all treated mice (Fig. S3). However, at 6 dpi, heterogeneous responses were detected between the Reg‐treated mice (Fig. S4). Although all mice in both the 4 dpi and 6 dpi groups survived (Fig. 1B), distinct differences in body weight emerged. While the 4 dpi group maintained near‐initial body weight, the 6 dpi group exhibited significantly lower weights. Furthermore, the Reg‐treated group can be divided into two populations: approximately 40% survived and 60% died. Therefore, as the experiment progressed towards the 10 dpi death point, we expected the surviving and dying populations within the 6 dpi group to exhibit significant differences in key molecular events, such as IFN and cytokine‐mediated signaling. These differences likely explain the variations observed in the Reg‐treated group at 6 dpi. Therefore, our analysis focused on the data from 4 dpi.

We validated RNA‐sequence (RNA‐seq) analysis for *Stat1*, *Stat2*, *Tnf‐α*, and *Ccl3* in these signaling pathways by RT‐qPCR. As shown in Fig. 2B, SARS‐CoV‐2 infection upregulated IFN‐stimulated *Stat1* and *Stat2* transcription factors, *Tnf‐α*, an inflammatory cytokine, and *Ccl3*, a chemokine of interstitial macrophages. Reg treatment subsequently reduced these levels, confirming its immunosuppressive effects (Fig. 2B). Upon IFN stimulation, STAT1 and STAT2 are phosphorylated by Janus kinase (JAK), leading to their translocation into the nucleus. Once in the nucleus, these phosphorylated STAT proteins elevate transcription levels of IFN target genes [16]. Notably, Reg treatment decreased the levels of *Stat1* and *Stat2*, which had been elevated by SARS‐CoV‐2 infection (Fig. 2B). This could explain the broad suppressive effect of Reg on IFN signaling. Collectively, RNA‐seq analysis provided strong evidence that Reg can mitigate COVID‐19 pathology by suppressing the early immune response through inhibition of IFN and cytokine signaling.

### Reg suppressed the activation of NLRP3 inflammasome in SARS‐CoV‐2‐infected mice

Activation of the inflammasome constitutes an essential part of the innate immune response. The inflammasome is composed of inflammasome sensors such as nucleotide‐binding oligomerization domain (NOD) and NOD‐like receptor (NLR) proteins, adaptor such as apoptosis‐associated speck‐like protein containing a CARD, and pro‐caspase‐1 [17]. We analyzed the alterations of these inflammasome components including pyroptosis genes at 4 dpi. When SARS‐CoV‐2 virus‐infected vs Reg‐treated groups were compared, a number of inflammasome sensor genes, including NLR family pyrin domain containing 3 *(Nlrp3)*, was significantly downregulated in the latter group, whereas *Nlrp10* was upregulated (Fig. 2C). Further, *Casp1* for caspase‐1 and its target *Gsdmd* for gasdermin D that contributes to pyroptosis by forming membrane pores, were also downregulated (Fig. 2C). We could also observe downregulation of *Casp3* and *Bak1* (Fig. 2C), suggesting the additional inhibitory effect of Reg on apoptotic cell death.

**Fig. 3:**
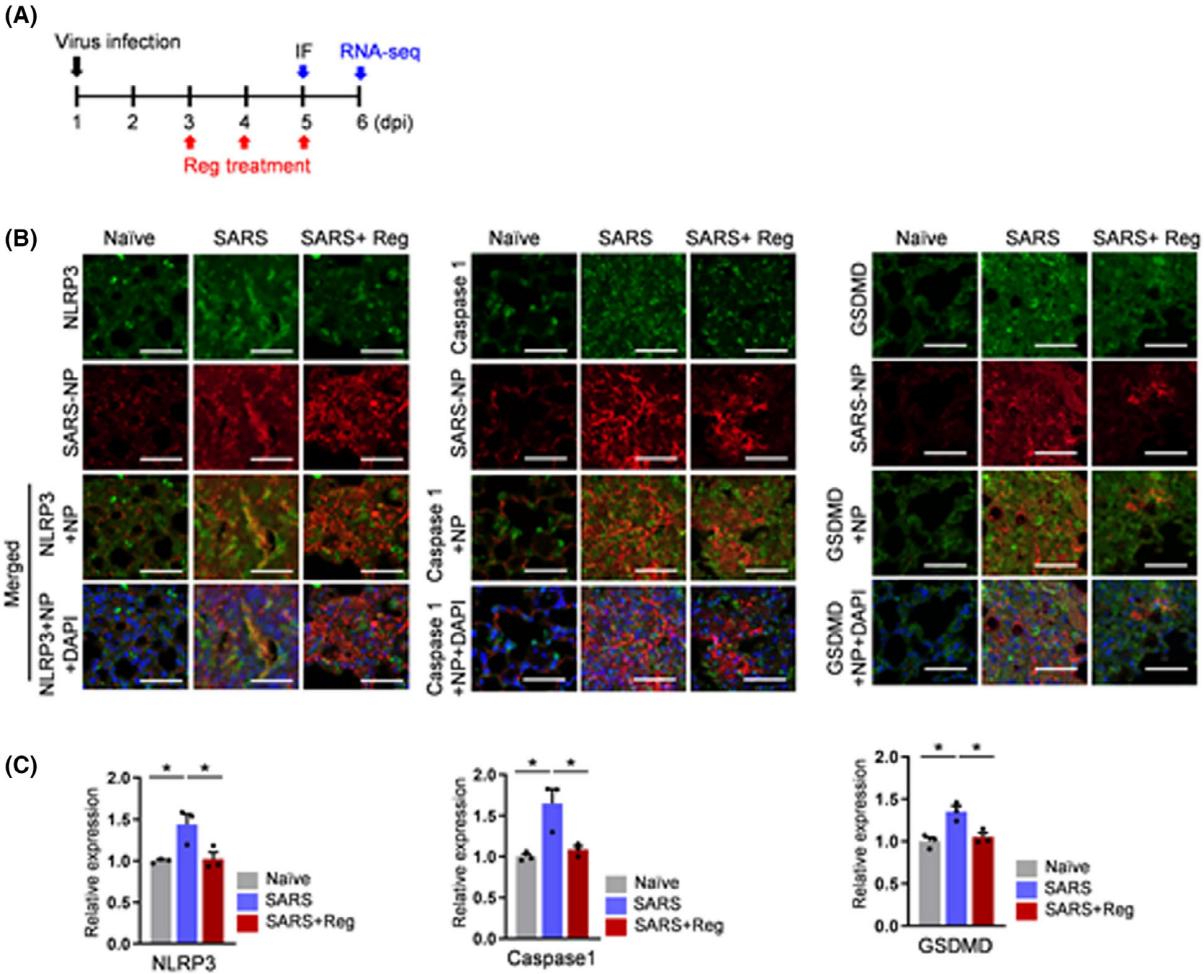
Validation for effect of regorafenib by immunofluorescence staining in SARS‐CoV‐2 infected lungs. (A) Experimental scheme for immunofluorescence (IF). (B) Representative lung immunofluorescence images for NLRP3, Caspase 1 and GSDMD. Mice were exposed to three different conditions: Naïve, SARS‐CoV‐2 virus, and SARS‐CoV‐2 combined with Reg. Lungs were harvested from the treated mice at 5 dpi, and subsequently analyzed by immunofluorescence. Scale bars, 100 μm. (C) Quantification of staining intensity. Naïve, non‐infected; SARS, SARS‐CoV‐2‐infected; SARS + Reg, Reg‐treated following SARS‐CoV‐2 infection (*N* = 3 for each group). Student's *t*‐test, *P < 0.05. Error bars represent standard error of the mean (SEM) for each group of mice.

To validate RNA‐seq analysis, we performed RT‐qPCR (Fig. 2D) and immunofluorescence (Fig. 3). NLRP3 is a well‐known inflammasome sensor that plays a crucial role in COVID‐19 pathogenesis [18, 19, 20]. Therefore, mRNA expression levels of *Nlrp3*, *Casp1* and *Gsdmd* in the *Nlrp3* inflammasome pathway were analyzed by RT‐qPCR. Expression levels for *Nlrp3* mRNA were elevated in SARS‐CoV‐2‐infected lungs and were responsive to Reg treatment, which likely contributed to the attenuation of inflammation in Reg‐treated mice (Fig. 2D). However, RT‐qPCR analysis of *Nod1*, NLR family CARD domain‐containing protein 4 (*Nlrc4*), *Nlrc5* and *Nlrp10* did not reach statistical significance, although it showed an expected trend, possibly due to their subtle alterations or individual variation (Fig. S5). Of downstream effectors of the *Nlrp3* inflammasome, *Casp1* and *Gsdmd* also displayed a similar response to Reg treatment (Fig. 2D). Consistent with these RNA‐seq data, co‐staining for NLRP3, caspase‐1 and GSDMD with viral NP exhibited a positive response, albeit with some variability, to Reg treatment (Fig. 3). Collectively, these results suggest that inhibiting excessive inflammasome activation can mitigate the cytokine storm, thereby improving the survival rate of Reg‐treated mice.

**Fig. 4:**
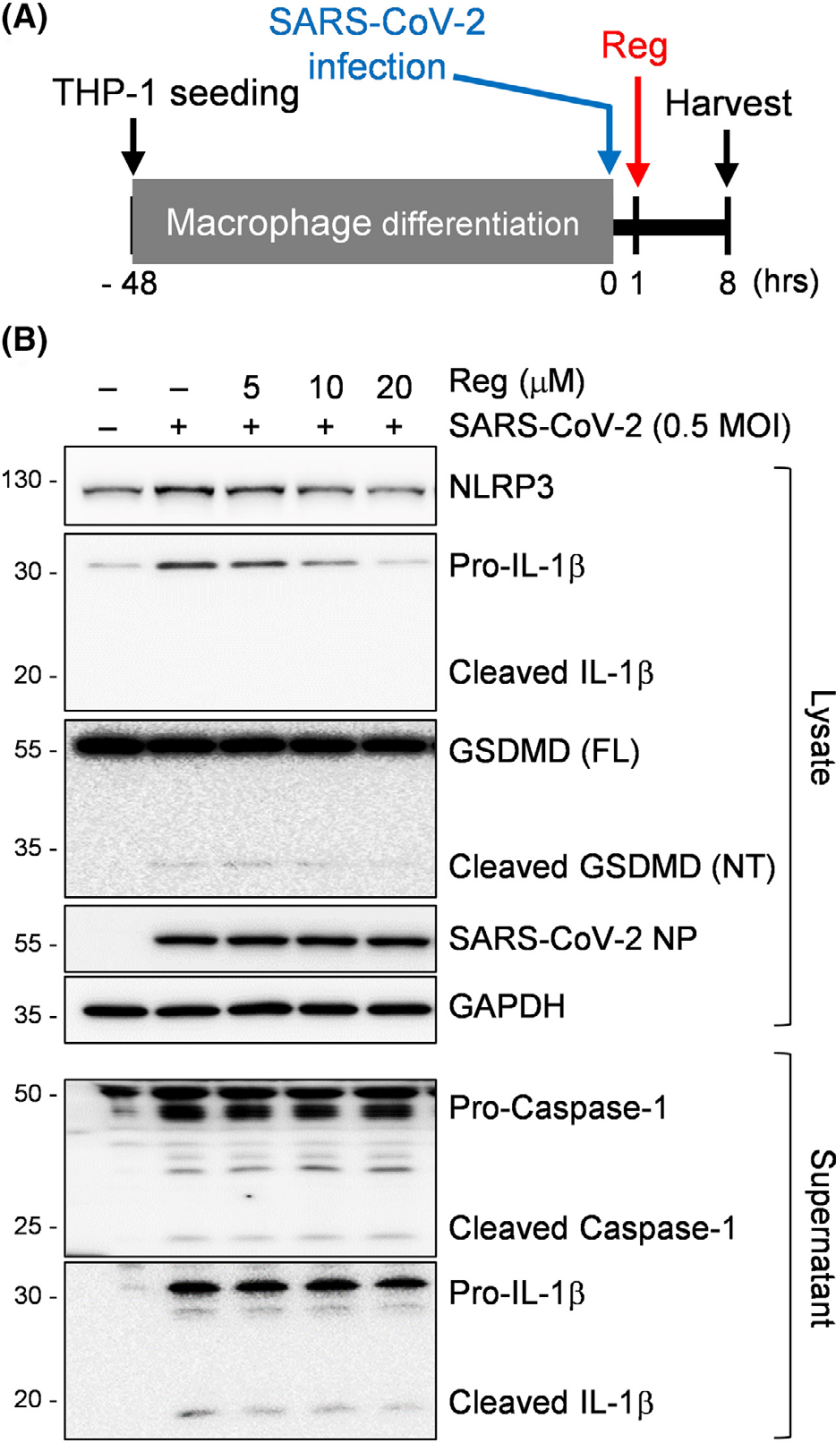
Effect of regorafenib on the NLRP3 inflammasome activation following SARS‐CoV‐2 infection. (A) Experimental scheme. THP‐1 monocytes were differentiated into macrophages by PMA (100 nm) incubation for 2 days, then infected with SARS‐CoV‐2 (0.5 MOI), treated with increasing concentrations of Reg, and finally harvested. (B) Cell lysates and supernatants were subjected to western blot analysis for the indicated proteins. Representative blots are shown from three independent experiments.

To further gain mechanistic insights into the Reg effect on the activation of NLRP3 inflammasome, we investigated alterations in its components in SARS‐CoV‐2 virus‐infected macrophages. SARS‐CoV‐2 virus were infected with or without Reg in THP‐1 cells. Although SARS‐CoV‐2 virus does not replicate in THP‐1 cell, a human macrophage cell line, virus exposure can induce increased pro‐inflammatory responses, such as production of TNF‐α, a transcriptional regulator of NLRP3 inflammasome [21, 22]. NLRP3 inflammasome activation triggers the cleavage of pro‐caspase‐1 into caspase‐1, which subsequently cleaves pro‐IL‐1β into IL‐1β and GSDMD at its N‐terminus, leading to pyroptotic cell death [23]. As depicted in Fig. 4A, SARS‐CoV‐2 virus‐infected macrophages were treated with Reg, and harvested. Western blot analysis of cell lysates revealed a dose‐dependent decrease in NLRP3 and Pro‐IL‐1β levels, but not GSDMD (Fig. 4B, top). While cleaved IL‐1β was not detected, a modest level of cleaved GSDMD was observed, suggesting that pyroptosis of infected macrophages may not be a prominent occurrence under these conditions. Consistent with this, cleaved caspase‐1 and IL‐1β was also secreted at low levels (Fig. 4B, bottom). Collectively, our macrophage experiments partially replicated the Reg effect on SARS‐CoV‐2 lung infection in mice. This could be due to the complex nature of lung tissue, which consists of diverse cell types, including macrophages.

Our study demonstrated that regorafenib inhibits the activation of the NLRP3 inflammasome, thereby suppressing SARS‐CoV‐2‐induced hyperinflammation and improving survival rates in hACE2 mice. Reg may modulate the NLRP3 inflammasome similarly to compounds such as MCC950, which effectively inhibits NLRP3 activation [24]. Moreover, colchicine has shown efficacy in reducing inflammation in COVID‐19 patients [25], while oridonin exhibits both antiviral properties and immune‐modulatory effects [26]. These findings underscore the therapeutic potential of targeting the NLRP3 inflammasome as a promising strategy for managing COVID‐19. Furthermore, the NLRP3 inflammasome regulates senescence and can promote fibrosis via pathways involving TGF‐β1 and epithelial‐mesenchymal transition [27, 28]. Given its ability to mitigate senescence [4] and lung fibrosis in the bleomycin model [29], Reg may also be effective in preventing the development of fibrosis, a serious complication, following COVID‐19 infection [30, 31]. In conclusion, drug repurposing of Reg may provide a therapeutic approach for treatment of severe COVID‐19.

## Conflict of interest

The authors declare no conflict of interests.

## Author contributions

JHJ, SOK, and SCM conducted and analyzed viral infection and biochemical experiments. EYS, MSS, and EGK interpreted the results and supervised the research. The paper was written by EGK and EYS. All authors have discussed the results, read and approved the final manuscript.

## Supporting information


**Fig. S1.** Immunofluorescence in SARS‐CoV‐2 infected lungs.
**Fig. S2.** Evaluation of the viral replications in lung tissues of SARS‐Cov‐2 infected K18‐hACE2‐tramsgenic mice.
**Fig. S3.** Heat maps for the modified immune response at 4 dpi.
**Fig. S4.** Heat maps for the modified immune response at 6 dpi.
**Fig. S5.** Validation of inflammasome sensors by RT‐qPCR.

## Data Availability

The data analyzed during this study are included in this published article and the supplemental data files. Additional supporting data are available from the corresponding authors upon reasonable request.
