# Supplementary material for: Regorafenib as a potential drug for severe COVID‐19: inhibition of inflammasome activation in mice
Source: FEBS Open Bio. 2025 Feb 3;15(3):427–35. doi: 10.1002/2211-5463.70002 (PMC11891780; doi:10.1002/2211-5463.70002)
Supplement: Supplementary file 1 — Fig. S1. Immunofluorescence in SARS‐CoV‐2 infected lungs. Fig. S2. Evaluation of the viral replications in lung tissues of SARS‐Cov‐2 infected K18‐hACE2‐tramsgenic mice. Fig. S3. Heat maps for the modified immune response at 4 dpi. Fig. S4. Heat maps for the modified immune response at 6 dpi. Fig. S5. Validation of inflammasome sensors by RT‐qPCR. [file FEB4-15-427-s001.docx]

**Supplementary Figures for**

**Regorafenib as a potential drug for severe COVID-19: inhibition of inflammasome activation in mice**

Ju Hwan Jeong^1, §^, Sun-Ok Kim^2, §^, Seong Cheol Min^1^, Eung-Gook Kim^2^, Min-Suk Song^1^ and Eun-Young Shin^2^

Department of Microbiology^1^ and Biochemistry^2^, Chungbuk National University College of Medicine and Medical Research Center, Chunbuk National University Hospital, Cheongju, 28644, Republic of Korea

^§^These authors contributed equally to this work.

* Co-corresponding authors:

Correspondence to: eyshin@chungbuk.ac.kr; songminsuk@chungbuk.ac.kr

**This PDF file includes:**

Fig. S1 to S5


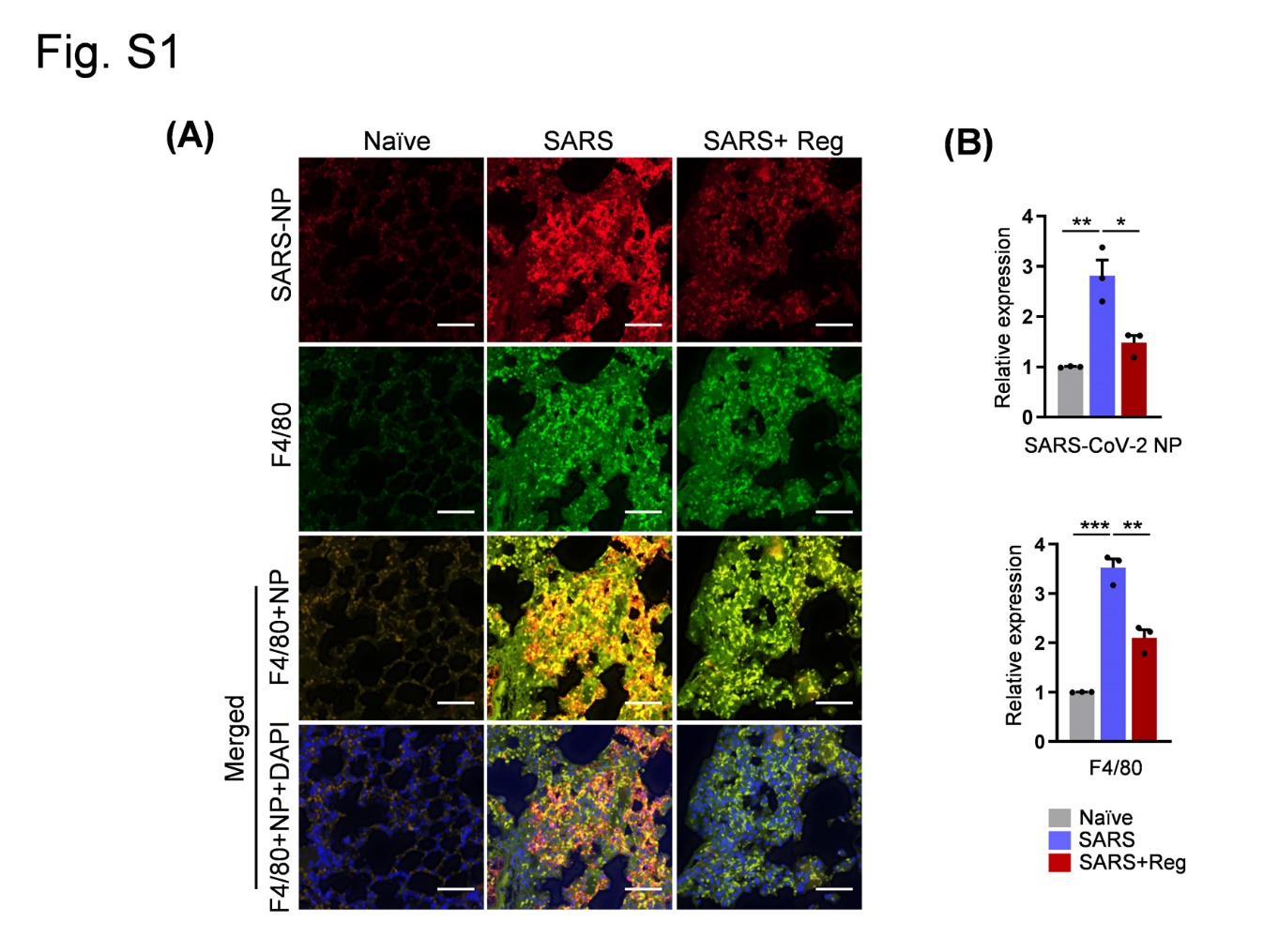


**Fig. S1 Immunofluorescence in SARS-CoV-2 infected lungs.**

(A) Representative lung immunofluorescence images for SARS-CoV-2 NP and F4/80. Mice were exposed to three different conditions: Naïve, SARS-CoV-2 virus, and SARS-CoV-2 combined with Reg. Lungs were harvested from the treated mice at 5 dpi, and subsequently analyzed by immunofluorescence. SARS-CoV-2 infection was confirmed by NP-positive staining. Scale bars, 100 μm. (B) Quantification of staining intensity of NP or F4/80. N=3 for each group. Student’s *t*-test, *, p < 0.05; **, p < 0.01; ***. p < 0.001. Error bars represent standard error of the mean (SEM) for each group of mice.


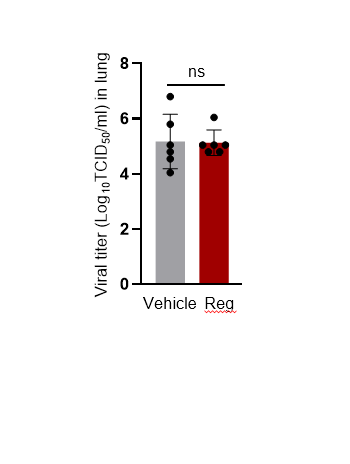


**Fig. S2 Evaluation of the viral replications in lung tissues of SARS-Cov-2 infected K18-hACE2-tramsgenic mice.**

Lung tissues of Reg-treated or Vehicle-treated mice were collected at 5 dpi (N=6). The viral titer is expressed in log_10_ TCID_50_/ml. Student’s t-test, ns, not significant. Error bars represent standard error of the mean (SEM) for each group of mice.


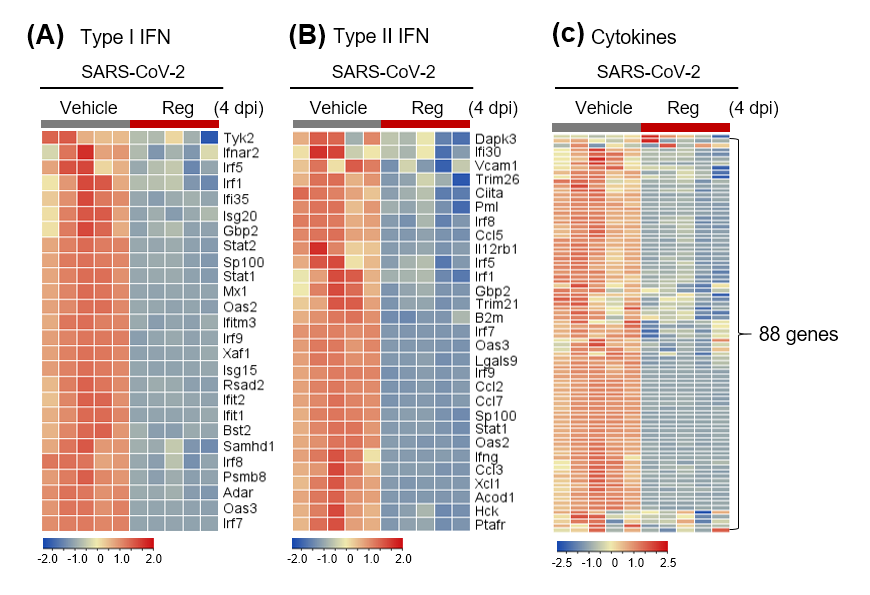


**Fig. S3 Heat maps for the modified immune response at 4 dpi.**

(A)-(C), Regorafenib-modulated genes in the type I (A) and II (B) IFN signaling pathway and cytokine-mediated signaling pathway (C) at 4 dpi following SARS-CoV-2 infection are shown. N=5 for each group. (C) Lists of altered cytokines; Trim26, Isg20, Rsad2, Irf9, Ifit2, Ifitm3, Xaf1, Pml, Adar, Isg15, Ifit1, Stat1, Oas3, Irf7, Mx1, Cxcl10, Tank, Ccl5, Bst2, Ifi35, Trim21, Stat2, Sp100, Psma7, Psmd4, Csf2ra, Tnfrsf18, Cd40, Ackr3, Tnf, Tnfsf9, Il21r, Ifi30, Psmb9, Tnfrsf8, Ifng, Il12rb1, B2m, Ubc, Osmr, Oas2, Lmnb1, Ciita, Xcl1, Psmb10, Ccl3, Psmc4, Tyk2, Ifnar2, Il2rg, Fas, Csf1, Ccl7, Psme2, Tnfrsf1b, Il2ra, Ccl2, Il18bp, Cxcl9, Gbp2, Samhd1, Irf5, Tnfrsf9, Pik3cd, Vcam1, Hck, Irf1, Psma5, Ptafr, Cxcl13, Pycard, Itgb2, Ptpn6, Birc5, Hmox1, Il2rb, Irf8, Il10ra, Syk, Itgam, Vav1, Timp1, Psmb6, Cfl1, Psmb8, Il1rn, Psme1, Nod1


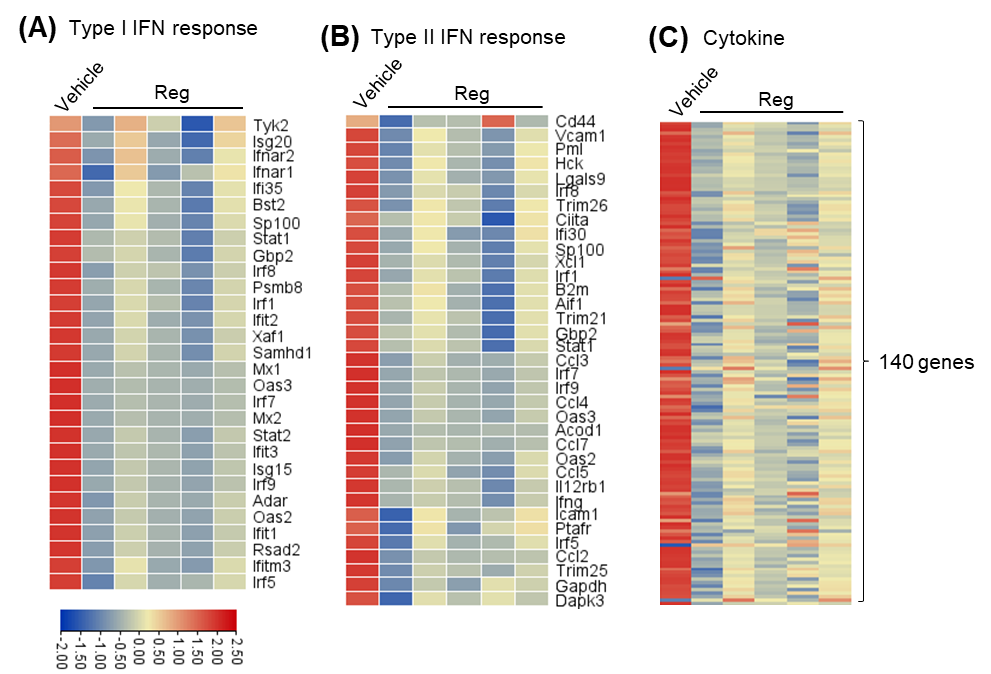


**Fig. S4 Heat maps for the modified immune response at 6 dpi.**

(A)-(C), Regorafenib-modulated genes in the type I (A) and II (B) IFN signaling pathway and cytokine-mediated signaling pathway (C) at 6 dpi following SARS-CoV-2 infection are shown. N=5 for each group. (C) Lists of altered cytokines; Trim26, Trim25, Mx2, Isg20, Rsad2, Irf9, Ifit2, Ifit3, Il12b, Ifitm3, Xaf1, Pml, Adar, Isg15, Ifit1, Stat1, Oas3, Irf7, Mx1, Cxcl10, Tank, Nod2, Cxcl11, Ccl5, Bst2, Ifi35, Trim21, Stat2, Sp100, Lama5, Lcn2, Fn1, Mmp2, Tnfrsf12a, Lrp8, Ccr1, Psma7, Il17ra, Psmd4, Csf2ra, Vim, Tnfrsf13b, Ereg, Tnfrsf1a, Tnfrsf18, Rorc, Cd40, Tnf, Tnfsf9, Il21r, Ifi30, Psmb9, Tnfrsf8, Ifng, Il12rb1, Tnfrsf4, Smarca4, B2m, Cd44, Ubc, Osmr, Oas2, Aim2, Stat4, Lmnb1, Ciita Xcl1, Relt, Ifnar1, Tnfrsf14, Psmb10, Hsp90b1, Ccl3, Psmc4, Tyk2, Ifnar2, Tnfrsf10b, Il2rg, Il3ra, Jagn1, Cd86, Gpr35, Csf1, Icam1, Ccl7, Psme2, Tnfrsf1b, Psmd3, Il2ra, Ccl2, Il18bp, Cxcl9, Gbp2, Samhd1, Irf5, Lcp1, Cd300lf, Tnfrsf9, Il27ra, Ccl4, Pik3cd, Vcam1, Hck, Irf1, Csf2rb, Jak3, Myc, Lif, Tnip2, Psma5, Ptafr, Pycard, Itgb2, Ptpn6, Birc5, Hmox1, Il2rb, Irf8, Il1r2, Ltbr, Il10ra, Syk, Mmp3, Ccr5, Inpp5d, Il15ra, Itgam, Vav1, Timp1, Psmb6, Cfl1, Socs3, Psmb8, Il1rn, Pim1, Psme1, Stat3, Nod1, Flrt3, Cdkn1a


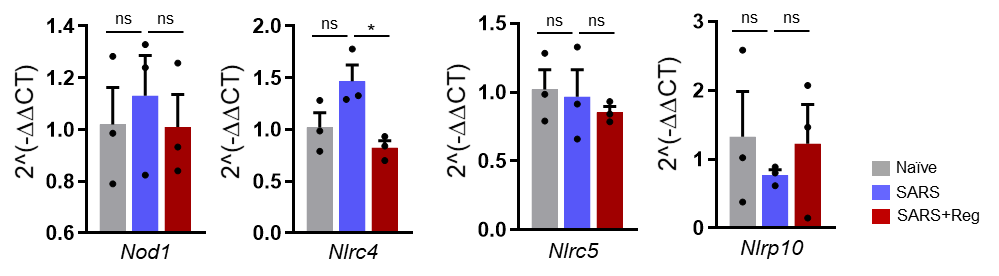


**Fig. S5 Validation of inflammasome sensors by RT-qPCR.**

RT-qPCR analysis was conducted to determine mRNA levels for *Nod1*, *Nlrc4*, *Nlrc5* and *Nlrp10*. No significant difference was observed between 3 groups. Naïve; SARS, SARS-CoV-2-infected; SARS + Reg, Reg-treated following SARS-CoV-2 infection. N for each group = 3. Student’s *t*-test, *, p < 0.05; ns, no significance. Error bars represent standard error of the mean (SEM) for each group of mice.
